# Silver(I) and Copper(II) 1,10-Phenanthroline-5,6-dione Complexes as Promising Antivirulence Strategy against *Leishmania*: Focus on Gp63 (Leishmanolysin)

**DOI:** 10.3390/tropicalmed8070348

**Published:** 2023-06-30

**Authors:** Simone S. C. Oliveira, Claudyane A. Correia, Vanessa S. Santos, Elaine F. F. da Cunha, Alexandre A. de Castro, Teodorico C. Ramalho, Michael Devereux, Malachy McCann, Marta H. Branquinha, André L. S. Santos

**Affiliations:** 1 Laboratório de Estudos Avançados de Microrganismos Emergentes e Resistentes (LEAMER), Departamento de Microbiologia Geral, Instituto de Microbiologia Paulo de Góes (IMPG), Universidade Federal do Rio de Janeiro (UFRJ), Rio de Janeiro 21941-901, Brazil; simonesantiagorj@yahoo.com.br (S.S.C.O.); claudyane.alves@ufrj.br (C.A.C.); vanessadssantos@ymail.com (V.S.S.); 2 Laboratório de Modelagem Molecular, Departamento de Química, Universidade Federal de Lavras, Lavras 37200-000, Brazil; elaine_cunha@ufla.br (E.F.F.d.C.); alexandre.a.castro@hotmail.com (A.A.d.C.); teo@ufla.br (T.C.R.); 3 The Centre for Biomimetic & Therapeutic Research, Focas Research Institute, Technological University Dublin, D08 CKP1 Dublin, Ireland; michael.devereux@tudublin.ie; 4 Chemistry Department, Maynooth University, W23 F2H6 Maynooth, Ireland; malachy.mccann@mu.ie; 5 Programa de Pós-Graduação em Bioquímica (PPGBq), Instituto de Química (IQ), Universidade Federal do Rio de Janeiro (UFRJ), Rio de Janeiro 21941-909, Brazil

**Keywords:** *Leishmania amazonensis*, alternative chemotherapy, antivirulence strategy, coordination compounds, gp63, virulence

## Abstract

Leishmaniasis, caused by protozoa of the genus *Leishmania*, encompasses a group of neglected diseases with diverse clinical and epidemiological manifestations that can be fatal if not adequately and promptly managed/treated. The current chemotherapy options for this disease are expensive, require invasive administration and often lead to severe side effects. In this regard, our research group has previously reported the potent anti-*Leishmania* activity of two coordination compounds (complexes) derived from 1,10-phenanthroline-5,6-dione (phendione): [Cu(phendione)_3_].(ClO_4_)_2_.4H_2_O and [Ag(phendione)_2_].ClO_4_. The present study aimed to evaluate the effects of these complexes on leishmanolysin (gp63), a virulence factor produced by all *Leishmania* species that plays multiple functions and is recognized as a potential target for antiparasitic drugs. The results showed that both Ag-phendione (−74.82 kcal/mol) and Cu-phendione (−68.16 kcal/mol) were capable of interacting with the amino acids comprising the active site of the gp63 protein, exhibiting more favorable interaction energies compared to phendione alone (−39.75 kcal/mol) or 1,10-phenanthroline (−45.83 kcal/mol; a classical gp63 inhibitor) as judged by molecular docking assay. The analysis of kinetic parameters using the fluorogenic substrate Z-Phe-Arg-AMC indicated V_max_ and apparent K_m_ values of 0.064 µM/s and 14.18 µM, respectively, for the released gp63. The effects of both complexes on gp63 proteolytic activity were consistent with the in silico assay, where Ag-phendione exhibited the highest gp63 inhibition capacity against gp63, with an IC_50_ value of 2.16 µM and the lowest inhibitory constant value (K_i_ = 5.13 µM), followed by Cu-phendione (IC_50_ = 163 µM and K_i_ = 27.05 µM). Notably, pretreatment of live *L. amazonensis* promastigotes with the complexes resulted in a significant reduction in the expression of gp63 protein, including the isoforms located on the parasite cell surface. Both complexes markedly decreased the in vitro association indexes between *L. amazonensis* promastigotes and THP-1 human macrophages; however, this effect was reversed by the addition of soluble gp63 molecules to the interaction medium. Collectively, our findings highlight the potential use of these potent complexes in antivirulence therapy against *Leishmania*, offering new insights for the development of effective treatments for leishmaniasis.

## 1. Introduction

Leishmaniasis is a group of neglected tropical diseases caused by protozoan parasites belonging to the *Leishmania* genus. Actually, more than 20 species of *Leishmania* have the capacity to be transmitted through female phlebotomine sand flies to humans [1,2]. *Leishmania* spp. have a dimorphic life cycle in which the extracellular flagellated promastigotes survive in the salivary glands of the sand fly vector until inoculation into the mammalian host, where they are phagocytosed by macrophages and then transform into intracellular amastigotes [1,2]. According to the World Health Organization (WHO), an estimated 0.7 to 1 million new cases occur annually and more than 1 billion people live in areas endemic for leishmaniasis with potential risk of developing the infection [3]. Leishmaniasis is a complex disease with three clinical manifestations: cutaneous leishmaniasis (CL), mucocutaneous leishmaniasis (MCL) and visceral leishmaniasis (VL). CL is the most common form of the disease, characterized by the appearance of ulcers and inflammatory lesions on the skin, which can produce permanent deformities in the infected individual, leading to social stigma. MCL, although rarer, is considered serious as it partially or totally destroys the mucous membranes of the nose, pharynx and throat. VL, also known as kala-azar, affects mainly the liver, spleen, lymph nodes and bone marrow, being potentially fatal if not properly treated [1,4]. The clinical manifestations of this illness depend on the host’s immune background and status [5]. For instance, *Leishmania amazonenis* is the etiologic agent of either localized or diffuse CL in Brazil; however, it has also been implicated in the etiology of VL in immunosuppressed patients, including those with hematologic disorders [6]. Another relevant issue is the phenotypical and genotypical backgrounds of the *Leishmania* isolates/species, which will drive the ability to express mechanisms of resistance as well as different and multiple virulence factors to cause more or less severe clinical manifestations [5].

Although various chemotherapy drugs are available for the treatment of leishmaniasis, such as pentavalent antimonials, amphotericin B deoxycholate (AmB), miltefosine, paromomycin and pentamidine, none of them are considered ideal due to their side effects, toxicity, high cost and long treatment time. Additionally, the emergence of drug resistance has become one of the greatest challenges in treating this parasitic disease [7,8,9]. In this context, the development of nanotechnology-based drugs has become an attractive approach for the treatment of leishmaniasis. Nanotechnology offers efficient and targeted drug delivery to specific tissues and cells, improving bioavailability and reducing toxicity [7,8,9]. Liposomes, nanoemulsions, polymeric nanoparticles and solid lipid nanoparticles are some examples of nanotechnology-based drug delivery systems used for the treatment of various infectious diseases [8]. Combination drug therapy is also being explored with the aim of identifying a treatment that is better tolerated and less prone to the development of resistance [7,9]. Recent studies have shown promising results in the topical treatment of cutaneous leishmaniasis using cationic nanovesicles loaded with miltefosine, meglumine antimoniate and imiquimod [10] as well as elastic nanovesicles (H-ENVs) coated with hyaluronate loaded with paromomycin, miltefosine and amphotericin B [11]. Nanotransfersomes loaded with miltefosine-polyphenol (apigenin) have also demonstrated a reduction in lesion size in previously infected mice [12]. Topical formulations have shown few adverse effects, making them a potentially promising therapy for the treatment of leishmaniasis [10,11]. The antivirulence strategy has gained interest in the international community as a promising alternative approach to combat microbial infections. It focuses on disabling virulence attributes that are essential for pathogens to develop the infectious process, without affecting microbial multiplication and without inducing resistance [13,14].

Virulence factors are components of infectious agents that cause damage to the host, consistently contributing to the establishment and maintenance of the infection [15]. *Leishmania* spp. possess an arsenal of virulence factors that allow parasites to infect, survive and replicate within the host cells, including a glycoprotein of 63 kDa named gp63 or leishmanolysin. Gp63 is a zinc-dependent metallo-type peptidase bound to the *Leishmania* plasma membrane by a glycosylphosphatidylinositol (GPI) anchor [16,17,18]. Gp63 is abundant in promastigote forms, whereas in intracellular amastigotes, its expression is down-regulated [17,19]. This protein is also secreted by the parasite, being released into the extracellular environment inside vesicles called exosomes or outside them [17,20,21]. This glycoprotein has been increasingly studied as an important virulence factor since it mediates important phases of the parasite infection, contributing to the initiation and maintenance of the infection through the modulation of macrophage functions in favor of parasite survival [17,18,21,22]. During the infectious process, gp63 participates in events such as (i) attachment of promastigotes to surface receptors expressed by host phagocytic cells, such as macrophages; (ii) inactivation of complement components and transcription factors; and (iii) degradation of host cytoskeletal proteins, nucleoproteins, extracellular matrix components and components involved in the intracellular signaling cascades [17,21,22]. The multifunctional roles played by gp63 turns this glycoprotein a promising target for drug development, since inhibition of this crucial virulence factor may disarm *Leishmania* cells and consequently reduce the damage to the host cells/tissues [16,20].

The *Leishmania* gp63 is a member of the metzincin family of zinc-metallopeptidases, and its hydrolytic activity is efficiently inhibited by 1,10-phenanthroline (phen) [16,18]. Our research group demonstrated that treating *L. amazonensis*, *L. braziliensis* and *L. infantum* with phen resulted in various biological alterations, such as irreversible morphological, morphometric, ultrastructural and physiological changes, leading to parasite death via apoptosis [23,24,25]. Additionally, phen decreased the infection rate of promastigotes in murine macrophages, with a clear involvement of gp63 in this crucial step of the infectious process. Interestingly, the pre-treatment of *L. braziliensis* promastigotes with sub-inhibitory concentrations of phen also significantly reduced their interaction with LL-5 cells, which derived from *Lutzomyia longipalpis*, which is a potential vector of this parasite [26] as well as both murine [23] and hamster [24] macrophages. However, exposure to high concentrations of phen can cause adverse effects on human health, such as skin and eye irritation, respiratory tract irritation and gastrointestinal distress [27]. With these premises in mind, the international chemistry scientific community has proposed synthesizing novel phen-derived compounds with better effectiveness and lower toxicity to multicellular organisms for potential applications in various contexts. For instance, 1,10-phenanthroline-5,6-dione (phendione), either in its metal-free state or coordinated with transition metals (e.g., silver and copper ions), was well-tolerated in vitro by different mammalian cell lines, including macrophages, as well as in vivo by *Galleria mellonella* larvae, Swiss mice [28] and hamsters [24]. Notably, both [Ag(phendione)_2_]ClO_4_ and [Cu(phendione)_3_](ClO_4_)_2_.4H_2_O complexes exhibited excellent protective action in *L. braziliensis* infections in hamsters, limiting the development of the paw lesion as well as drastically reducing the number of parasites in either infected paw or lymph nodes draining the lesion, suggesting their high potential as new, safe and effective alternatives for leishmaniasis treatment [24].

In the present study, the anti-gp63 activity of both [Ag(phendione)_2_]ClO_4_ and [Cu(phendione)_3_](ClO_4_)_2_.4H_2_O was evaluated using in silico and in vitro approaches, aiming to use these potent complexes in the future as platform to support and develop antivirulence strategies against *Leishmania*.

## 2. Materials and Methods

### 2.1. Test Compounds

1,10-Phenanthroline-5,6-dione (phendione), [Ag(phendione)_2_]ClO_4_ (Ag-phendione) and [Cu(phendione)_3_](ClO_4_)_2_.4H_2_O (Cu-phendione) were synthetized according to the methods previously described in the literature [29]. The compounds were dissolved in dimethyl sulfoxide (DMSO; Sigma-Aldrich, St. Louis, MO, USA) and stored at room temperature in the dark. All other reagents were of analytical grade.

### 2.2. In Silico Analyses

A molecular docking study was carried out to investigate the interaction modes between the *Leishmania* gp63 protein and the following test compounds: phen (a classical inhibitor of gp63), phendione and its silver(I) and copper(II) derivatives. In this way, it was possible to unravel the interaction energies (in kcal/mol) resulting from the docking of these compounds into the gp63 active site, thus obtaining their most stable conformations. The compounds used in this work were constructed and optimized by semi-empirical methods in the PC Spartan *Pro* program, more precisely at the PM3 level. The partial charges for the constituent atoms in their structures were also calculated. For the docking simulations, the Molegro Virtual Docker (MVD^®^; Molexus IVS, Rørth, Denmark) program [30] was used, in which each ligand was docked in the gp63 active site. In this step, the identification of the ligand interaction modes was interactive, evaluating a number of solutions (conformation and orientation of the ligand) and estimating the energy of their interactions with the enzyme. For each ligand, 50 runs were carried out, with each run returning a solution (pose). The water molecules were kept flexible in order to obtain a system closer to the real environment. A radius of 5 Å was considered at the enzyme active site to carry out the calculations. The crystal structure of gp63 was obtained from the Protein Data Bank (PDB), under the code 1LML [31].

For the enzyme preparation, the ions and water molecules were removed from the original PDB, with the exception of water molecules that were in the active site. The addition of polar hydrogen atoms was performed according to the protonation state of the receptor at pH 7.4, using the PDB2PQR tool (https://server.poissonboltzmann.org/pdb2pqr; accessed on 5 May 2023). Generally, lower energy values stand for better protein–ligand interactions; that is, these interactions are more stabilizing. Thus, the molecular docking technique determines the most likely conformation of the ligand in the enzyme active site [30]. To find the lowest energy structure, without any previous assumptions, it is necessary to analyze all interaction modes, by considering the conformational flexibility of the ligand to be introduced into the active site. However, the number of combinations involved is very large [32,33,34]. The values of the Docking Scoring Function, Score, are defined by Equation (1):

(1) $$E_{\mathrm{score}}=E_{\mathrm{inter}}+E_{\mathrm{intra}}$$

wherein:

(2) $$E_{\mathrm{inter}}=\sum_{\mathrm{i\varepsilon ligand}}\sum_{\mathrm{j\varepsilon protein}}\left[E_{\mathrm{PLP}}(r_{\mathrm{ij}})+332.0\frac{\mathrm{qiqj}}{4r_{\mathrm{ij}}^{2}}\right]$$

*E_inter_* is the ligand–protein interaction energy. *E_PLP_* (Equation (2)) stands for “piecewise linear potential”, which consists of the use of two different sets of parameters, as described forward: one for approximation of the steric term (Van der Waals) among atoms, and the other potential for the hydrogen bonding. The second term is related to the electrostatic interactions among overloaded atoms. It is a Coulomb potential with a distance-dependent dielectric constant (D(r) = 4r). The numerical value of 332.0 is responsible for the electrostatic energy unit to be given in kilocalories per molecule (kcal mol^−1^) [30].

*E_intra_* is the internal energy of the ligand:

(3) $$E_{\mathrm{intra}}=\sum_{\mathrm{i\varepsilon ligand}}\sum_{\mathrm{j\varepsilon ligand}}E_{\mathrm{PLP}}(r_{\mathrm{ij}})+\sum_{\mathrm{flexiblebonds}}A\left[1-\cos\left(m.\theta -\theta_{0}\right)\right]+E_{\mathrm{clash}}$$

The first part of the equation (double summation) is among all pairs of atoms in the ligand, taking off those that are connected by two bonds. The second one characterizes the torsional energy, where *θ* is the torsional angle of the bond. If several torsions could be determined, each torsional energy is considered and there is the use of an average among them. The last term, *E_clash_*, assigns a penalty of 1000 if the distance between two heavy atoms (more than two bonds apart) is smaller than 2.0 Å, not taking into account infeasible ligand conformations [30]. The docking search algorithm that is applied in the MVD program considers an evolutionary algorithm, interactive optimization techniques which are inspired by Darwinian evolution theory, and a new hybrid search algorithm called guided differential evolution. This hybrid combines the differential evolution optimization technique with a cavity prediction algorithm during the search process, allowing a fast and accurate identification of potential binding modes (poses) [30,33,34]. 

### 2.3. Parasite and Growth Conditions

*Leishmania amazonensis* strain (MHOM/BR/PH8) was supplied by Coleção de *Leishmania* from Fundação Oswaldo Cruz—FIOCRUZ (Instituto Oswaldo Cruz, Rio de Janeiro, Brazil, *Leishmania* Type Culture Collection—LTTC-WDCM 731). For the experiments, promastigote forms were grown in Schneider’s medium (Sigma-Aldrich, St. Louis, MO, USA), pH 7.2, supplemented with 10% fetal bovine serum (FBS; Cultilab, São Paulo, Brazil) at 26 °C for 72 h. Parasites were harvested by centrifugation at 4000× *g* during 10 min at 4 °C and then washed three times in phosphate-buffered saline (PBS; 150 mM NaCl, 20 mM phosphate buffer, pH 7.2). The number of promastigotes was estimated by counting them in a Neubauer chamber. 

### 2.4. Secretion Assay to Obtain the Gp63-Rich Supernatant

*L. amazonensis* promastigotes (10^9^ cells) were resuspended in 2 mL of sterile isotonic PBS supplemented with 2% glucose and incubated for 3 h at both 26 °C (invertebrate host temperature) and 37 °C (mammalian host temperature). After this interval, parasite cells were removed by centrifugation and the supernatant (named PBS-glucose-conditioned supernatant) was passed over a 0.22-μm sterilizing membrane (Millipore, São Paulo, Brazil). The protein concentration was quantified by the method described by Lowry et al. [35], using bovine serum albumin (BSA) as standard. In parallel, the survivability and the morphology of *L. amazonensis* promastigotes during the incubation period in PBS-glucose was assessed by (i) direct observation of the parasite motility through light microscopic analysis; (ii) Giemsa staining of parasites to detect nucleus and kinetoplast; (iii) propidium iodide staining in order to measure, by flow cytometry, the uptake level of this compound by damaged parasite cells due to a loss in plasma membrane integrity; and (iv) flow cytometry analysis, in order to measure two morphological parameters, cell size and granularity. In some of these experiments, parasites were treated with 0.4% paraformaldehyde or sodium azide (0.95 g/L) for 30 min in order to obtain non-viable cells to use as a negative control in the viability tests. 

### 2.5. Secreted Protein Profile

PBS-glucose-conditioned supernatant (equivalent to 10 μg protein) was added to gel electrophoresis sample buffer (125 mM Tris, pH 6.8, 4% SDS, 20% glycerol and 0.002% bromophenol blue) and mixed with 5% 2-β-mercaptoethanol, followed by heating at 100 °C for 5 min. Proteins were analyzed in 12% sodium dodecyl sulfate-polyacrylamide gel electrophoresis (SDS-PAGE) by the method described by Laemmli [36]. Electrophoresis was carried out at 4 °C, at 120 V, and the gels were silver stained. Molecular mass of sample polypeptides was calculated from mobility of molecular mass standards (Thermo Fisher Scientific, Waltham, MA, USA). 

### 2.6. Western Blotting for Detecting the Gp63 Protein

Proteins present in PBS-glucose-conditioned supernatant were separated in 12% SDS-PAGE and then electrophoretically transferred at 4 °C at 100 V/300 mA for 2 h to a nitrocellulose membrane. The membrane was blocked in 5% low-fat dried milk dissolved in Tris-buffered saline (TBS; 150 mM NaCl; 10 mM Tris, pH 7.4) containing 0.1% Tween 20 (TBS/Tween) for 1 h at room temperature. The membrane was washed three times (10 min each) with the blocking solution and incubated for 2 h with the primary anti-gp63 antibody (raised against the recombinant gp63 molecule from *L. mexicana* and kindly provided by Peter Overath of the Max-Planck-Institut für Biologie, Abteilung Membranbiochemie, Germany) at 1:1000 dilution. After this period, the membrane was washed three times with TBS-Tween 0.1% and then incubated with the secondary antibody peroxidase-conjugated goat anti-rabbit IgG at 1:25,000. Finally, the membrane was washed again with the same buffer and revealed by chemiluminescence detection after reaction with ECL immunoreagents and analyzed by ImageQuant™ LAS 4000. The molecular mass of each protein was calculated by comparing it to molecular mass standards (Thermo Fisher Scientific, Waltham, MA, USA).

### 2.7. Zymography for Evidencing the Gp63 Proteolysis 

Proteolytic activity was assayed by SDS-PAGE containing 0.1% gelatin (Sigma-Aldrich, St. Louis, MO, USA) incorporated into the gel as the proteinaceous substrate [37]. Briefly, 10 micrograms of proteins present in the PBS-glucose-conditioned supernatant were added to the SDS-PAGE sample buffer (125 mM Tris, pH 6.8, 4% SDS, 20% glycerol and 0.002% bromophenol blue) and subsequently subjected to electrophoresis, which was processed at 200 V/20 mA, for 2 h at 4 °C. Subsequently, SDS was removed by incubation of the gels with 2.5% Triton X-100 (Sigma-Aldrich, St. Louis, MO, USA) for 1 h at room temperature under constant agitation. The gels were incubated for 48 h at 37 °C in 50 mM glycine-NaOH buffer, pH 10.0, to promote proteolysis [38] in the absence and in the presence of classical proteolytic inhibitors as follows: 10 mM phen, 10 mM phenylmethylsulphonyl fluoride (PMSF; Sigma-Aldrich, St. Louis, MO, USA) and 10 µM *trans*-epoxysuccinyl L-leucylamido-(4-guanidino) butane (E-64; Sigma-Aldrich, St. Louis, MO, USA). Finally, the gels were stained with 0.2% Coomassie Brilliant Blue R-250 (Sigma-Aldrich, St. Louis, MO, USA) in methanol–acetic acid–water (50:10:40) and destained in a solution containing methanol–acetic acid–water (5:10:85). The molecular masses of the peptidases were estimated by comparison with the mobility of molecular mass standards (Thermo Fisher Scientific, Waltham, MA, USA). 

### 2.8. Detection of Gp63 Activity Using Fluorogenic Substrate

The enzymatic activity of secreted gp63 was evaluated by the cleavage of the fluorogenic substrate Z-Phe-Arg-AMC. A 5 mM stock solution of the fluorogenic substrate was prepared in DMSO. The reaction was initiated by adding different substrate concentrations (2.5 µM, 5.0 µM, 10 µM and 20 µM) to gp63 (20 µg protein) in a total volume of 100 µL of 50 mM glycine-NaOH buffer, pH 10.0 [38]. At the same time, the titration of the amount of gp63 was also performed. In this sense, different concentrations of gp63 (ranging from 0.156 to 20 µg) were added to 10 µM Z-Phe-Arg-AMC substrate in a total volume of 100 µL of 50 mM glycine-NaOH buffer, pH 10.0. Cleavage of Z-Phe-Arg-AMC fluorogenic substrate was continuously monitored in a spectrofluorometer (SpectraMax Gemini XPS, Molecular Devices, Silicon Valley, CA, USA) using an excitation wavelength of 380 nm and an emission wavelength of 460 nm. The reaction mixture was incubated at 37 °C for 1 h and the assays were controlled for self-release of the fluorophore in the same time interval. Proteolytic activity was expressed as fluorescence arbitrary units (FAU). Apparent K_m_ and V_max_ values were calculated using a computerized non-linear regression from the data obtained from the Michaelis–Menten equation (GraphPad Prism 8). 

### 2.9. Effects of Ag-Phendione and Cu-Phendione on Gp63 Proteolysis

The effects of both Ag-phendione and Cu-phendione on modulating gp63 proteolysis over the fluorogenic substrate Z-Phe-Arg-AMC were evaluated. To perform this, the reaction mixture containing 10 μg protein, 10 µM substrate and 50 mM glycine-NaOH buffer, pH 10.0, was incubated in the absence or presence of different concentrations of Ag-phendione (ranging from 40 to 0.625 μM) and Cu-phendione (ranging from 1000 to 15.62 μM). Fluorescence was measured using a SpectraMax Gemini XPS fluorescence microplate reader (Molecular Devices, Silicon Valley, CA, USA) at an excitation wavelength of 380 nm and an emission wavelength of 460 nm. The drug concentration capable of reducing 50% (IC_50_) of gp63 activity compared to the control was calculated after 1 h of reaction by linear regression of absorbance versus drug concentration, using the Excel program. The inhibitory constant (K_i_) value was calculated using a derivation of the Michaelis–Menten equation employing GraphPad Prism 8. 

### 2.10. Effects of Ag-Phendione and Cu-Phendione on Stability of Binding to Gp63

The effects of Ag-phendione and Cu-phendione on the stability of binding to gp63 were evaluated using gelatin-SDS-PAGE as previously reported [39,40]. In this method, a gp63 solution (containing 10 µg) was incubated in the absence or in the presence of different concentrations of the test compounds ranging from 0.39 to 50 µM for Ag-phendione and 3.9 to 500 µM for Cu-phendione for 1 h at 37 °C. Then, gel electrophoresis sample buffer was added to the reactional mixtures, which were subjected to electrophoresis analysis by gelatin-SDS-PAGE. The densitometric analysis of the proteolytic bands was performed using the ImageJ program. 

### 2.11. Effects of Ag-Phendione and Cu-Phendione on Parasite Cells: Looking for Gp63 Levels

In this set of experiments, *L. amazonensis* promastigotes (1 × 10^6^ cells) were cultured for 24 h at 26 °C in the absence (control) or presence of either IC_50_ or 2 × IC_50_ values of both Ag-phendione (IC_50_ for 72 h = 7.8 nM) and Cu-phendione (IC_50_ for 72 h = 7.5 nM) as previously reported by our group [25]. After that, promastigotes were washed and processed to evaluate both gp63 activity and gp63 protein levels. 

For gp63 proteolytic activity, initially, whole parasite cellular extracts were obtained by repeated freeze/thawing cycles using 10^6^ viable cells in PBS containing 1% CHAPS (3-[(3-cholamidopropyl) dimethylammonium]-1-propanesulfonate), pH 7.0. Then, the cellular extract was centrifuged at 10,000× *g* for 10 min at 4 °C, and the supernatant was used to quantify the protein content by the method described by Lowry et al. [35]. The gp63 proteolytic activity was determined as previously described using the cleavage of the fluorogenic substrate Z-Phe-Arg-AMC. 

For gp63 protein detection, untreated and treated promastigotes with both test compounds were fixed with 0.4% paraformaldehyde in PBS (pH 7.2) for 10 min or permeabilized with 4% paraformaldehyde in PBS (pH 7.2) for 30 min, both at room temperature. Then, the parasite cells were incubated with a 1:200 dilution of anti-gp63 antibody for 1 h at room temperature. Cells were then incubated for an additional hour with a 1:100 dilution of fluorescein isothiocyanate (FITC)-labeled goat anti-rabbit immunoglobulin G (IgG) (Sigma-Aldrich). Finally, parasites were washed three times with PBS and analyzed by flow cytometry (FACSCalibur, BD Bioscience, San Jose, CA, USA) equipped with a 15-mW argon laser emitting up to 488 nm. PBS-treated cells were run in parallel as controls. The data represented the analysis of 10,000 events, and the results were expressed as percentage of fluorescent cells.

### 2.12. Effects of Ag-Phendione, Cu-Phendione and Soluble Gp63 on Promastigotes–Macrophage Interaction

The human leukemia monocytic cell line (THP-1) was maintained in 25-cm^2^ tissue culture flasks with RPMI 1640 medium (Sigma-Aldrich, St. Louis, MO, USA) supplemented with 10% FBS at 37 °C in an atmosphere containing 5% CO_2_. To perform interaction experiments, THP-1 cells were seeded in 24-well plates at a density of 2 × 10^5^ cells/well and then differentiated into macrophages by treatment with phorbol-12-myristate-13-acetate (PMA; 40 ng/mL) (Sigma-Aldrich) for 48 h. Then, the plates were washed twice with sterile PBS (pH 7.2) to remove PMA and a fresh RPMI 1640 medium was added. At the same time, *L. amazonensis* promastigotes were treated with the IC_50_ or 2 × IC_50_ values of the test compounds for 24 h. After this period, the parasites were allowed to infect the mammalian cells (10 parasites per macrophage) for 24 h at 37 °C and 5% CO_2_, in the absence or presence of 10 µg soluble gp63. Then, the cultures were washed with PBS to remove non-internalized parasites, fixed with methanol and stained with Giemsa. The percentage of infected macrophages was determined by randomly counting 200 cells on each of triplicate coverslips using a bright-field microscope. The association index was obtained by multiplying the percentage of infected macrophages by the number of amastigotes per macrophage.

### 2.13. Statistics

All experiments were performed in triplicate in three independent experimental sets. The data were analyzed statistically by means of Student’s *t* test using GraphPad Prism 8 software (GraphPad Software, San Diego, CA, USA). Values of *p* equal or less than 0.05 were considered statistically significant.

## 3. Results and Discussion

### 3.1. Ag-Phendione and Cu-Phendione Bind to the Gp63 Active Site: In Silico Approach

A 3D box-based cavity prediction algorithm, using the MVD program, was utilized to generate the gp63 binding sites. Among the several binding sites identified, the active cavity had a volume of 157.7 Ǻ^3^. According to the theoretical results of molecular docking (Table 1), Ag-phendione exhibited the lowest energy value of −74.82 kcal/mol, which is the most stabilizing interaction with the enzyme, followed closely by Cu-phendione (−68.16 kcal/mol). In contrast, phendione displayed the least favorable interaction energy with a value equal to −39.75 kcal/mol. Based on the theoretical docking results, the following order of interaction with gp63 can be observed: Ag-phendione > Cu-phendione > phen > phendione (Table 1).

**Figure 1:**
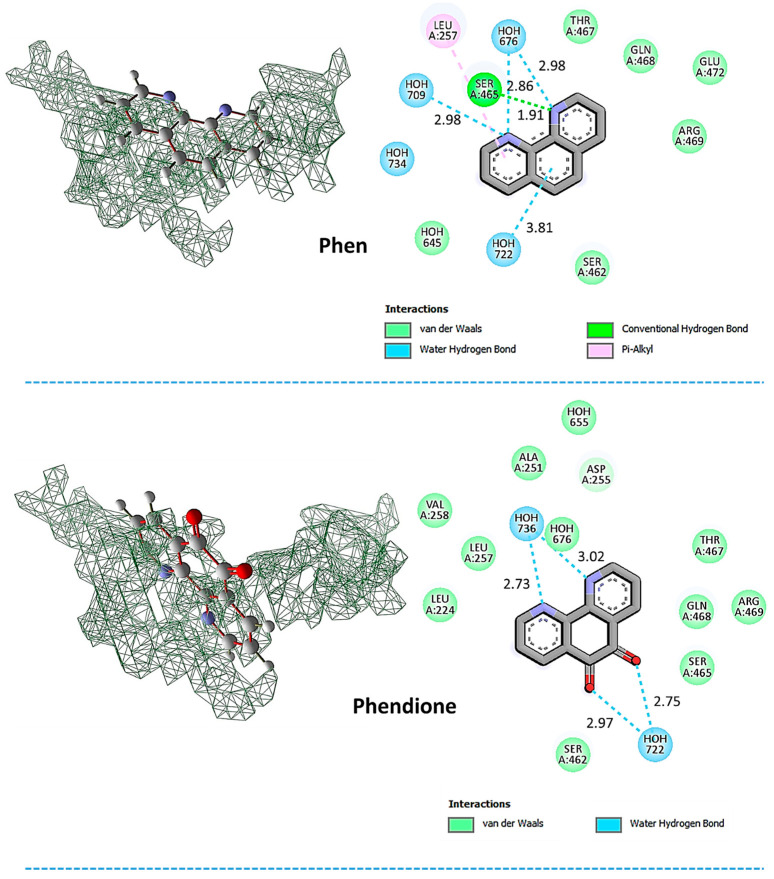

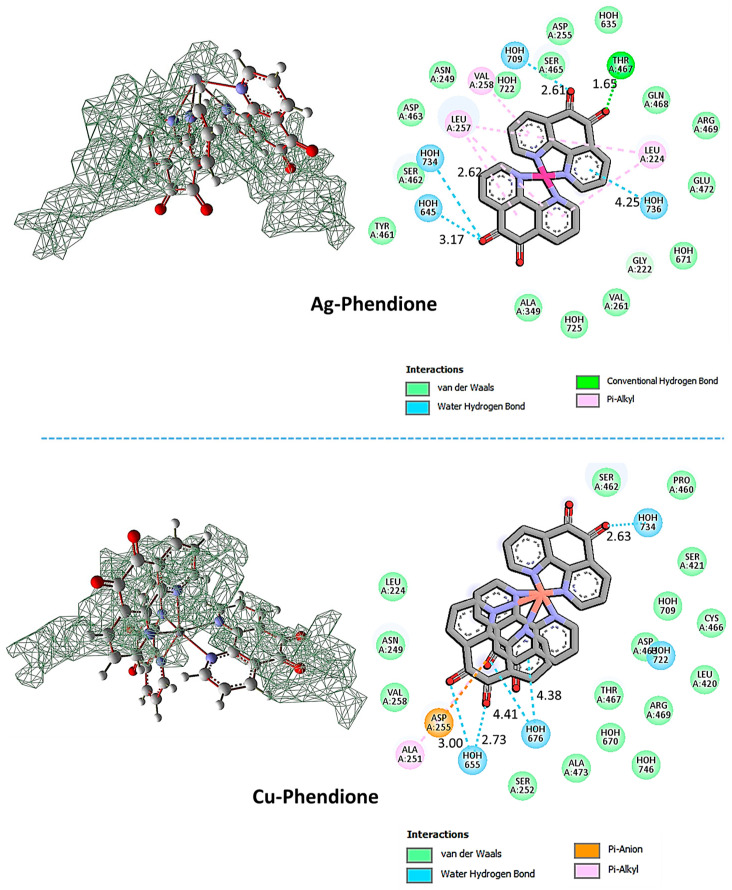
Representation of the test compounds docked into the active cavity (**left**) and intermolecular interactions (**right**) with *Leishmania* gp63 protein.

Figure 1 displays the docking results of the test compounds within the gp63 active site, along with their respective interaction modes. Upon observing Figure 1, it is evident that Ag-phendione and Cu-phendione, due to their bulkiness, occupied a significant portion of the active cavity, which may account for their more favorable intermolecular interaction energy compared to phen and phendione. Ag-phendione exhibited the highest number of interactions, including engagement with the Thr_467_ residue, four water molecules through hydrogen bonds, as well as multiple long-distance interactions (Van der Waals) and hydrophobic interactions. These intermolecular interactions play a crucial role in stabilizing the ligand within the active cavity. Furthermore, due to its conformation, Ag-phendione formed a π-stacking interaction between its rings, enabling strong interaction with the gp63 protein. This interaction involved diverse hydrogen bonds with water molecules and an amino acid residue, contributing to its more favorable interaction energy compared to the other test compounds. Similarly, Cu-phendione demonstrated a high number of interactions, involving various long-distance interactions and hydrogen bonds with three water molecules. Although it did not form hydrogen bonds with amino acid residues, the significant number of interactions led to a stable accommodation of the compound at the binding site. It is noteworthy that the bulkiness of Ag-phendione and Cu-phendione allowed them to interact with multiple residues in the binding site, enhancing the stability of the interaction with the enzyme.

Compared to the other compounds, phen formed four hydrogen bonds with Ser_465_ and three water molecules, along with some long-distance interactions. In contrast, phendione exhibited the least favorable interaction energy at the gp63 active site, which may be attributed to its limited interaction within the cavity. This observation is supported by the fact that phendione interacted with a smaller number of residues and formed hydrogen bonds solely with water molecules present in the cavity.

In a similar approach to our work, Galdino et al. [34] reported that phendione-based compounds were able to interact with the active site of the metallopeptidase LasB of the Gram-negative bacterium *Pseudomonas aeruginosa*. In that study, the docking analysis revealed that Cu-phendione adhered firmly to LasB active site via π-stacking interactions between aromatic rings, in addition to the greatest interaction energy (−127.54 kcal/mol), followed by phendione (−40.47 kcal/mol) and Ag-phendione (−34.26 kcal/mol). Furthermore, Cu-phendione was able to form two hydrogen bonds with amino acid residues Arg_198_ and Asn_112_ present in the LasB catalytic site, besides two hydrogen bonds with water molecules. Ag-phendione interacted with LasB through three water molecules and a hydrogen bond with the amino acid residue Ala_113_. As verified in the present work, phendione interacted weakly with the active site of the LasB enzyme, forming only one hydrogen bond with the amino acid Arg_198_ and another with one water molecule [34]. These results highlight the ability of both Ag-phendione and Cu-phendione to bind to relevant metallopeptidases of medically relevant microorganisms, which opens the possibility to use them as drug candidates in antivirulence strategies.

### 3.2. Secretion of Gp63 by L. amazonensis Promastigotes under Starvation Conditions

**Figure 2:**
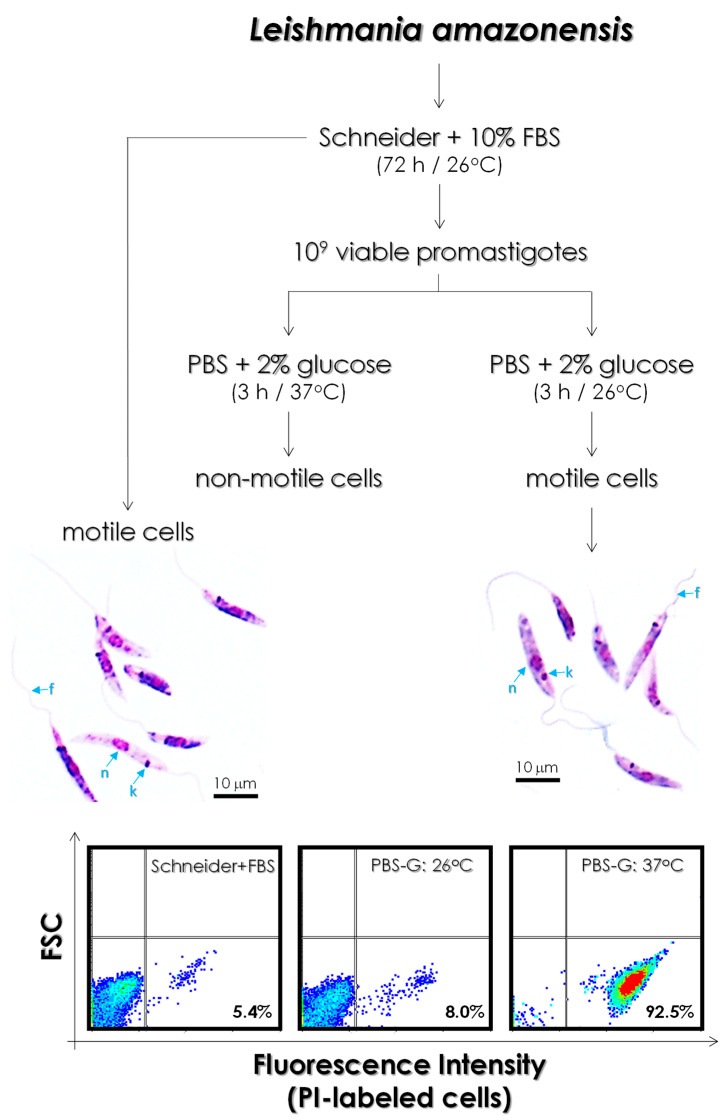
*Leishmania amazonensis* promastigotes were cultured for 72 h in Schneider’s medium supplemented with 10% fetal bovine serum at 26 °C. Subsequently, the equivalent to 10^9^ cells were collected and incubated in PBS-glucose (PBS-G) 0.1% at 37 °C (human temperature) or 26 °C (insect vector temperature) for 3 h. The morphology of the parasites was evaluated using Giemsa-stained smears and then analyzed under an optical microscope. Note the typical elongated promastigote form (n, nucleus; k, kinetoplast; f, flagellum). Plasma membrane integrity was analyzed by flow cytometry using propidium iodide (PI). Density plots show fluorescence intensity versus forward scatter (FSC, which represents the parasite size). The numbers in the bottom represent the percentage of PI-labeled cells (non-viable parasites).

In this set of experiments, we aimed to establish an easy protocol to obtain gp63 molecules to be used in the kinetic assays and to test the efficacy of both Ag-phendione and Cu-phendione complexes. Our idea was to evaluate the capacity of living parasites to secrete gp63 molecules in a chemically defined medium free of all the components present in complex media such as brain heart infusion and liver infusion tryptose. Based on this premise, *L. amazonensis* promastigotes were incubated under starving conditions (PBS supplemented with glucose) at two distinct temperatures (26 °C and 37 °C), and the parasite viability was assessed after 3 h of incubation. At the temperature mimicking the insect vector (26 °C), promastigotes maintained their characteristic morphology as judged by Giemsa staining, including the elongated cell body, single long flagellum, central nucleus and the position of the kinetoplast anterior to the nucleus as well as their size (FSC parameter) as observed by flow cytometry. Parasite cells presented motility in fresh preparations as well as viability since more than 90% of cells did not incorporate propidium iodide (Figure 2). Although maintaining their classical morphology and morphometry, promastigotes incubated in PBS-glucose at 37 °C lost their motility and showed intense passive incorporation of propidium iodide (92.5%) (Figure 2), a DNA intercalating agent that is excluded by viable cells but can penetrate cell membranes of dying or dead cells.

**Figure 3:**
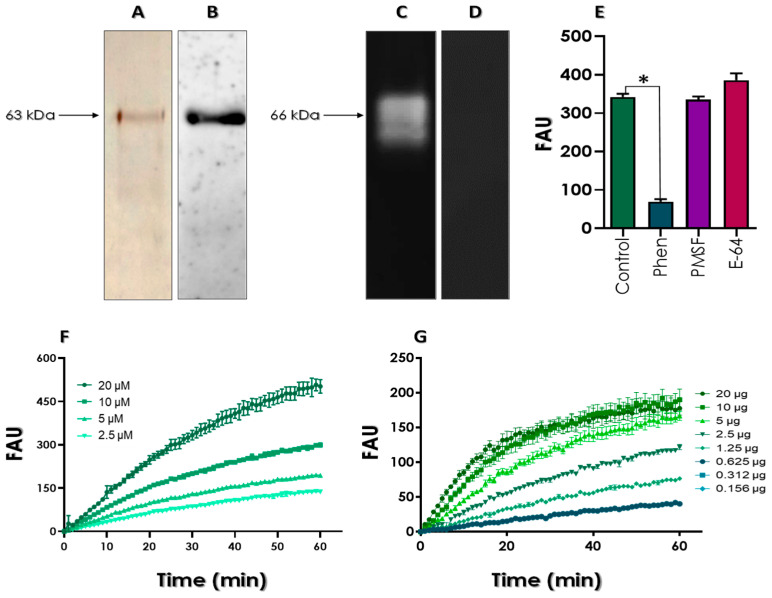
Detection of gp63 molecules in the PBS-glucose-conditioned supernatant of *L. amazonensis*. (**A**) SDS-PAGE showing the presence of a 63 kDa protein. (**B**) Western blotting evidencing a 63 kDa protein recognized by the anti-gp63 antibody. (**C**) The peptidase profile was analyzed by gelatin-SDS-PAGE revealing at least two proteolytic bands when the gel was incubated in 50 mM glycine-NaOH buffer (pH 10.0) at 37 °C for 48 h. (**D**) The metallopeptidase inhibitor phen at 10 mM blocked the gp63 enzymatic activity. (**E**) Analysis of the hydrolysis of the Z-Phe-Arg-AMC substrate by gp63 in the absence (control) or presence of 10 mM phen, 10 mM PMSF and 10 µM E-64. Results are expressed as fluorescence arbitrary units (FAU). The asterisks denote significant differences compared to the control (*p* < 0.05). (**F**) Analysis of the hydrolytic activity of gp63 using different concentrations of the fluorogenic substrate Z-Phe-Arg-AMC (2.5, 5, 10 and 20 µM). (**G**) Cleavage of the Z-Phe-Arg-AMC (10 µM) substrate by different gp63 protein concentrations (0.156–20 µg). The kinetics were performed in 50 mM glycine-NaOH buffer, pH 10.0, at 37 °C for 1 h.

Subsequently, the secretion content of *L. amazonensis* promastigotes was analyzed after 3 h of incubation under these starving conditions. SDS-PAGE analysis indicated the presence of a main single protein band of apparent molecular mass of 63 kDa (Figure 3A). In this regard, gp63 can be either anchored to the membrane via a GPI anchor or secreted directly into the extracellular medium in its glycosylated form. Due to the variations in the glycosylation state, the secreted gp63 can have different molecular mass ranges, varying from 60 to 70 kDa [17,41]. Gp63 homologues have also been detected in monoxenic trypanosomatids, in which different isoforms were identified, with molecular masses ranging from 50 to 97 kDa [41]. 

Western blotting analysis showed that the major protein on *L. amazonensis* secretion cross-reacted with the anti-gp63 antibody, confirming its immunogenicity (Figure 3B). It is well-known that gp63 is involved in the process of parasite survival and evasion of the host immune response [17]. In addition, gp63 is a critical antigen capable of leading to the production of antibodies by the infected patient, being a common immunogenic determinant that can be used in serodiagnosis assays for leishmaniasis [42]. Furthermore, gp63 is also being projected as a potential antigen for vaccine development [43].

The proteolytic activity of the secreted gp63 protein was firstly evaluated by gelatin-SDS-PAGE assay. Interestingly, the zymography revealed the presence of two major proteolytic bands around 66 kDa (Figure 3C), which were inhibited by 10 mM phen, a classical metallopeptidase inhibitor (Figure 3D). These two bands may correspond to different isoforms of gp63 that are secreted by *Leishmania*. The proteolytic activity analysis through SDS-PAGE with incorporated gelatin is a non-denaturing methodology since the samples are neither treated with denaturing agents nor heated. Therefore, protein denaturation is limited, so that dimer formation cannot be excluded and the two observed bands could also be related to dimeric forms of gp63 [37,39]. 

In order to confirm that the identified activity corresponds to the secreted metallopeptidase gp63, its ability to hydrolyze the Z-Phe-Arg-AMC substrate was evaluated at alkaline pH in the absence or presence of different proteolytic inhibitors. After 1 h of reaction, a strong inhibition of the proteolytic activity was verified only in the presence of phen, while PMSF (a serine peptidase inhibitor) and E-64 (a cysteine peptidase inhibitor) did not significantly change the enzymatic activity, confirming the presence of only metallo-type peptidases in the *L. amazonensis* PBS-glucose-conditioned supernatant (Figure 3E).

Finally, the kinetic parameters were investigated after titration of either Z-Phe-Arg-AMC substrate (Figure 3F) or gp63 protein amount (Figure 3G), showing in both cases a typical concentration-dependence of the proteolysis rate. The kinetic parameters calculated for gp63 over cleavage of Z-Phe-Arg-AMC substrate were: V_max_ = 0.064 µM/s and apparent K_m_ = 14.18 µM.

### 3.3. Effects of Test Compounds on the Enzymatic Activity of Gp63

**Figure 4:**
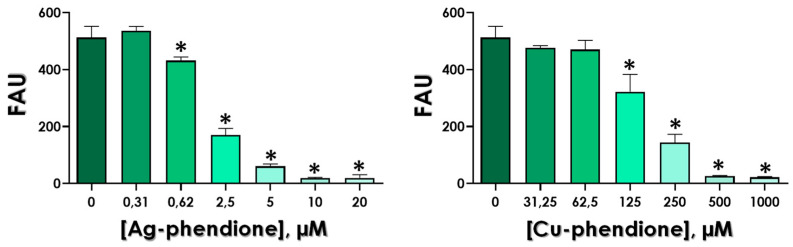
Effects of Ag-phendione and Cu-phendione on the proteolytic activity of *Leishmania* gp63. Gp63 (10 µg) was incubated in the absence or in the presence of different concentrations of Ag-phendione (ranging from 0.31 to 20 µM) or Cu-phendione (31.25 to 1000 µM). The Z-Phe-Arg-AMC substrate hydrolysis was evaluated after 1 h of reaction in 50 mM glycine-NaOH buffer, pH 10.0, at 37 °C. Results are expressed as fluorescence arbitrary units (FAU). The asterisks denote significant differences compared to the control (*p* < 0.05).

In this set of experiments, the test compounds were used to evaluate their efficacy in inhibiting the gp63 activity over the Z-Phe-Arg-AMC fluorogenic substrate. The data revealed a typical dose-dependent reduction in the substrate hydrolysis by both complexes, Ag-phendione and Cu-phendione (Figure 4). Corroborating the in silico analysis, Ag-phendione showed the greatest capacity to inhibit the secreted gp63 activity, presenting the lowest IC_50_ value (IC_50_ = 2.16 µM), as well as the lowest inhibitory constant value (K_i_ = 5.13 µM), followed by Cu-phendione (IC_50_ = 163 µM and K_i_ = 27.05 µM) (Table 2).

Gp63 is a metallopeptidase that can be located inside the parasite, anchored to the cell membrane or be released into the extracellular environment. It is an essential virulence factor for *Leishmania* spp., both in the vertebrate host and in the insect vector, and the inhibition of gp63 proteolytic activity can block fundamental pathways for the adaptation and survival of the parasite in its different hosts, being, therefore, an interesting target for the development of new bioactive compounds [18,22]. In this sense, our group previously verified that the activity of *L. braziliensis* gp63, located at either the parasite surface or secreted into the extracellular medium, was strongly inhibited by compounds derived from phendione in a dose-dependent manner. Furthermore, Cu-phendione also showed a more potent effect on the inhibition capacity of the membrane-associated form, reducing the proteolytic activity by 65% and 80% after treatment with 0.1 μg/mL and 1 μg/mL, respectively. Ag-phendione and phendione promoted an inhibition of 68% and 67%, respectively, at a concentration of 1 µg/mL. In addition, the activity identified in the culture supernatant was completely abolished in the presence of 1 μg/mL of all test compounds [44].

Galdino and collaborators [34] verified that phendione and its silver and copper derivatives were also capable of inhibiting the activity of LasB from *P. aeruginosa*; however, Cu-phendione presented the best inhibitory action on LasB (K_i_ = 0.09 µM), followed by Ag-phendione (K_i_ = 0.31 µM) and phendione (K_i_ = 0.38 µM). Similarly, in this work, we also found that the phendione ligand had significantly higher kinetic values than the complexes.

For several years, phen has been used as a platform for the synthesis of new compounds with antimicrobial action, among them phendione, which is synthesized from the inclusion of *N*-atoms in the phenanthrene ring and which has a considerable antimicrobial activity [28,45]. The structural difference between phendione and phen is that the former has two carbonyl groups at positions 5 and 6 of its central ring [28,46]. It is believed that the bioactivity of metal-free *N,N′*-chelating bases such as phen and phendione is related to their ability to sequester trace metals from the biological environment, with the resulting metal complexes being the real active species [28]. In this sense, the complexation of Ag^+^ and Cu^2+^ ions to the phendione ligand was able to increase its bioactive potential against different microorganisms, such as *L. amazonensis*, *L. infantum*, *L. brazilinsis* and *P. aeruginosa* [25,34,44].

### 3.4. Assessing the Stability of Gp63/Test Compounds

**Figure 5:**
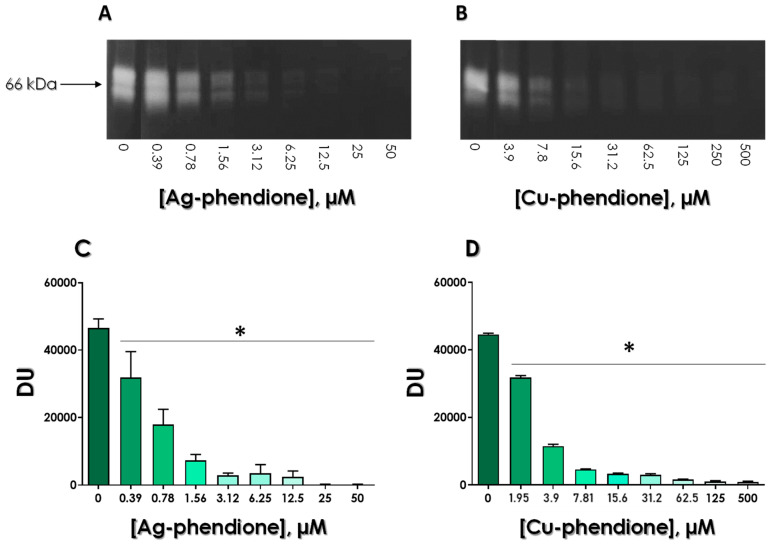
Effects of Ag-phendione and Cu-phendione on *Leishmania* gp63 inhibition stability. Gp63 (10 µg) was incubated with concentrations ranging from 50 to 0.39 µM of Ag-phendione (**A**) and 500 to 0.39 µM of Cu-phendione (**B**) for 1 h at 37 °C. Subsequently, the mixtures were analyzed by gelatin SDS-PAGE. The gels were incubated in glycine-NaOH buffer, pH 10.0, for 48 h at 37 °C and then stained with Coomassie blue. The number on the left indicates the apparent molecular mass, expressed in kilodaltons. (**C**,**D**) Densitometric analysis of proteolytic halos observed in gelatin-SDS-PAGE. The asterisks denote significant differences compared to the control (*p* < 0.05).

The gelatin-SDS-PAGE technique is suitable for studying the ability of peptidases to form stable complexes with their respective peptidase inhibitors [39,40]. To perform this, gp63 molecules were incubated with different concentrations of Ag-phendione and Cu-phendione for 1 h at 37 °C in order to form complexes between the peptidase and each test compound, which were then analyzed by gelatin-SDS-PAGE. As expected, the inhibition of gp63 activity was detected in a classical dose-dependent fashion, as judged by both gel visualization (Figure 5A,B) and densitometrical analyzes of the proteolytic bands (Figure 5C,D). Furthermore, it was clearly observed that the highest concentrations of the test compounds form stable complexes with gp63, blocking its hydrolytic activity even after electrophoresis (Figure 5).

SDS is an anionic detergent that acts as a denaturing agent capable of disrupting the three-dimensional structure of proteins due to the cleavage of non-covalent bonds, such as hydrogen bonds, hydrophobic interactions and ionic bond interactions. On the other hand, covalent bonds, such as disulfide bridges, remain unaffected by the presence of the detergent [47]. In this regard, the stability of complexes between peptidases and their inhibitors after SDS-PAGE has been claimed to indicate covalent bond formation, as proposed for cysteine peptidase (papain)/cystatin [40] and serine peptidase (human tissue plasminogen activator)/PAI-1 [39]. The stability of the Ag-phendione/gp63 and Cu-phendione/gp63 complexes during the electrophoresis is probably associated with the maintenance of enzyme-substrate complexes promoted by covalent bonds with amino acid residues. The binding stability is consistent with the low K_i_ value obtained for these complexes, which indicates a high specificity and, consequently, a greater capacity for the enzyme-inhibitor complex to remain stable, making it difficult to restore the proteolytic activity of gp63. The stability of papain/cysteine peptidase inhibitors [human stefin A (HSA), human stefin B (HSB), oryzacystatin I (OCI) and oryzacystatin I1 (OCIT)] complexes was evaluated by mildly-denaturing gelatin-polyacrylamide gel electrophoresis. The cysteine peptidase inhibitors HSA and HSB presented reduced K_i_ values, while OCI and, mainly, OCII presented high K_i_ values, indicating a greater affinity of HSA and HSB with papain, while OCII presented the lowest affinity for the enzyme. Corroborating these results, the analysis using mildly-denaturing gelatin-PAGE did not identify any proteolytic activity of the HSA/papain and HSB/papain complexes, when compared with the untreated control, confirming the greater stability of these complexes. On the other hand, the stability of the OCI/papain and OCII/papain complexes were directly affected during electrophoresis since a restoration of proteolytic activity was verified. OCI/papain complex showed a relative stability, inhibiting about 80% of the enzymatic activity; on the other hand, OCII restored almost 100% of the proteolytic activity of papain, which is in agreement with its high calculated K_i_ value [40].

### 3.5. Effects of Test Compounds on Gp63 Expression by Living L. amazonensis Promastigotes

Recently, our research group published a report demonstrating the excellent in vitro activity of both Ag-phendione and Cu-phendione against *L. amazonensis* promastigotes, displaying IC_50_ values at the nanomolar range (7.8 nM and 7.5 nM, respectively, calculated after 72 h of parasite-drug contact). Moreover, these complexes triggered the apoptosis-like death in *L. amazonensis* [25]. Parasite proliferation relies on their ability to acquire nutrients from various sources, and enzymes, including peptidases, play crucial roles in this vital process [48]. In this context, gp63 is directly involved in the nutritional capability of living *Leishmania* cells [48]. Based on these premises, promastigotes were treated with both test complexes at their IC_50_ and 2 × IC_50_ values for 24 h. Under these experimental conditions, the promastigotes maintained their viability and morphology. Subsequently, the parasites were processed for the detection of gp63 using flow cytometry and enzymatic measurement using the Z-Phe-Arg-AMC substrate.

**Figure 6:**
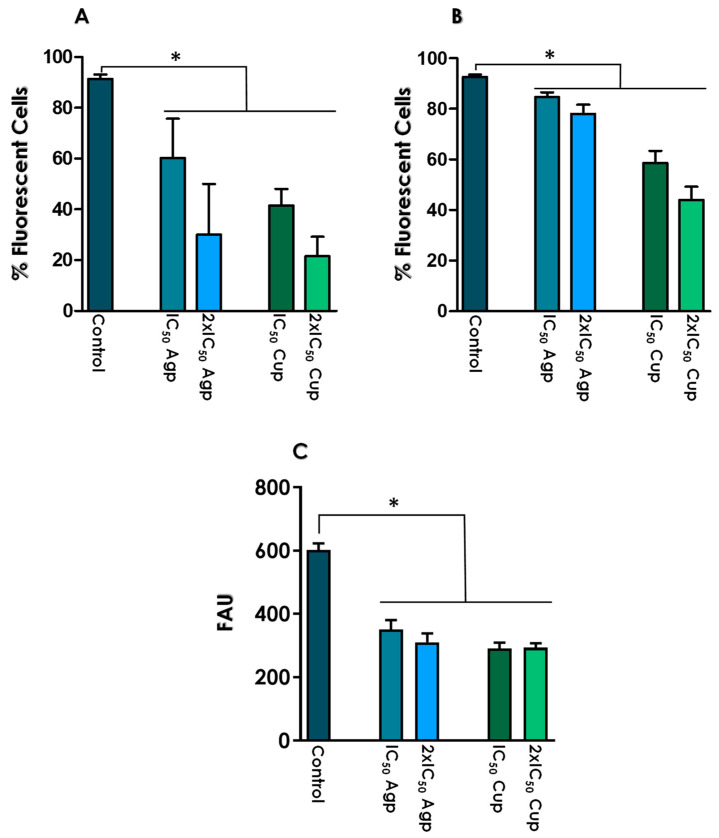
Detection of gp63 molecules in *L. amazonenis* promastigotes cultured in the absence (control) or in the presence of the IC_50_ and 2 × IC_50_ values of Ag-phendione (Agp) and Cu-phendione (Cup). Promastigotes, either fixed (**A**) or fixed and permeabilized (**B**), were processed for flow cytometry analysis using anti-gp63 antibody. Data are expressed as the percentage of fluorescent cells and represent the analysis of 10,000 cells. (**C**) Detection of Z-Phe-Arg-AMC substrate cleavage by *L. amazonensis* cell lysates cultured in the absence (control) or in the presence of the IC_50_ and 2 × IC_50_ values of Ag-phendione and Cu-phendione. Data represent the 1 h reaction point at 37 °C in glycine-NaOH buffer, pH 10.0. Results are expressed as fluorescence arbitrary units (AFU) and values represent the mean ± standard deviation of three independent experiments. The asterisks denote significant differences compared to the control (*p* < 0.05).

Flow cytometry analysis demonstrated that treatment with the test compounds resulted in a significant and dose-dependent reduction in the number of gp63-positive promastigotes, impacting both the cell surface (fixed parasites) (Figure 6A) and intracellular (fixed and permeabilized parasites) (Figure 6B) gp63 isoforms. In parallel, parasites treated with the test compounds exhibited a decreased ability to hydrolyze the Z-Phe-Arg-AMC substrate compared to untreated cells. This inhibitory effect was observed at both concentrations used. Specifically, treatment with Ag-phendione reduced the proteolytic capacity of cell-associated metallo-type peptidases to approximately 55%, while Cu-phendione decreased enzymatic activity to approximately 48% (Figure 6C).

It is already well studied that the proteolytic activity of gp63 bound to the surface of promastigotes is essential to increase the interaction of parasites with macrophages. In the early stages of the infectious process, gp63 is capable of inhibiting complement-mediated lysis, since the metallopeptidase interacts with the C3 component of the complement system cascade and cleaves C3b and C3bi, preventing the formation of the membrane attack complex and, consequently, facilitating phagocytosis by the macrophage [17,22]. The presence of gp63 also facilitates the attachment of *Leishmania* to the macrophage through fibronectin receptors. Gp63 has a SRYD sequence that is antigenically related to the fibronectin RGDS sequence, suggesting a probable interaction of gp63 with fibronectin receptors present on macrophages [20,49]. Previously, our group identified the presence of a secreted protein of approximately 63 kDa in the culture supernatant of avirulent and virulent promastigotes of *L. braziliensis,* displaying cross-reactivity with the anti-gp63 antibody, with a strong proteolytic activity that is completely inhibited by phen. The virulent strain secretes a higher level of gp63 proteins compared to the avirulent strain, which directly impact in the ability of these strains to interact with macrophages [44]. The proteolytic capacity of gp63 also plays an essential role on the cleavage of host extracellular matrix components, providing a faster internalization of *Leishmania* in the macrophage [17,22]. The reduced expression of gp63 strongly interferes with the establishment and maintenance of the infectious process since this metallopeptidase is responsible for reducing the host cell’s immune response [17,22]. In this sense, our results revealed that the pre-treatment of promastigotes with Ag-phendione and Cu-phendione diminished the expression and proteolytic activity of gp63, which can directly impact the interaction process with host cells.

### 3.6. Effects of Test Compounds on the Interaction with THP-1 Macrophages

**Figure 7:**
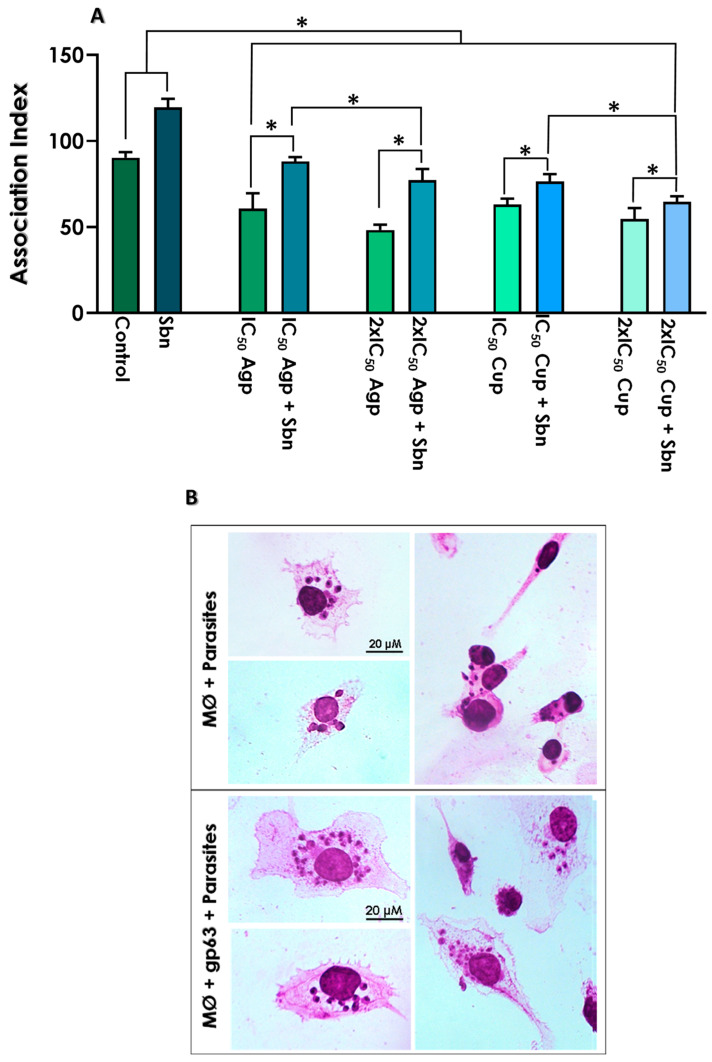
Effects of Ag-phendione, Cu-phendione and soluble gp63 on *L. amazonensis* promastigotes-macrophage (MØ) interaction. *L. amazonensis* promastigotes were placed to interact with THP-1 cells in the presence (Sbn) or absence (control) of 10 µg of the gp63-rich supernatant for 24 h. In parallel, *L. amazonensis* promastigotes were pre-treated or not (control) with the IC_50_ and 2 × IC_50_ values of Ag-phendione and Cu-phendione for 24 h and subsequently placed to interact with THP-1 cells in the presence or absence of 10 µg of the gp63-rich supernatant for 24 h. (**A**) The association indexes were determined by light microscopy, counting at least 200 cells in each of triplicate coverslips. The asterisks denote significant differences compared to the control (*p* < 0.05). (**B**) Bright-field microscopy of infected THP-1 cells in the presence or not of the gp63-rich supernatant.

Gp63 is found abundantly both on the cell surface and inside the parasite, being secreted into the extracellular medium in a free form or via exosomes and later internalized by macrophages, where they facilitate the survival of *Leishmania* in the host cell [19]. Herein, we evaluated the relevance of gp63 in the initial stages of the parasite-host interaction process. Initially, in order to confirm the importance of gp63 during the interaction process, THP-1 macrophage cells were placed to interact with *L. amazonensis* promastigotes in the presence or absence of the gp63-rich supernatant. Our data indicated a considerable increase (~34%) in the association index (AI) when the interaction was performed in the presence of the gp63-rich supernatant (AI = 119.6) compared to control systems without soluble gp63 (AI = 90.3) (Figure 7A,B).

Once determined that the complexes inhibited the expression and activity of gp63, our next step was to verify whether the pre-treatment of the promastigotes with Ag-phendione and Cu-phendione should affect the process of parasite–host interaction in the absence and presence of the gp63-rich supernatant (Figure 7A). Parasites pre-treated with the test compounds showed a reduced ability to interact with macrophages when compared with the untreated control. After treatment of promastigotes with the IC_50_ and 2 × IC_50_ values of Ag-phendione, AIs were calculated as 60.6 and 48.2, respectively. This effect was partially reversed when the interaction was evaluated in the presence of the gp63-rich supernatant, in which AI was enhanced to 88.1 and 77.3, respectively (Figure 7A). Similarly, *L. amazonensis* pretreated with the IC_50_ and 2 × IC_50_ values of Cu-phendione showed an AI of 63.1 and 54.8, respectively; in the presence of the gp63-rich supernatant, the infection capacity was partially restored, with the calculated AI raised to 76.5 for the parasites treated with the IC_50_ value and to 64.6 when the parasites were treated with the 2 × IC_50_ value of Cu-phendione (Figure 7A). Furthermore, treatment with the 2 × IC_50_ values of both compounds, in the presence of the gp63-rich supernatant, showed a lower ability to reverse the inhibitory action when compared to treatment with the IC_50_ values, as verified by the lowest AIs (Figure 7A).

The observed data showed that Ag-phendione and Cu-phendione were able to block the initial stages of the interaction process, with the former showing a subtle higher efficacy. In another study of our group, *L. braziliensis* promastigotes pre-treatment for 1 h with Ag-phendione and Cu-phendione resulted in a significant reduction in the number of amastigotes inside murine macrophages. Similar to the results verified in this work, Ag-phendione was the most potent compound, reducing the interaction process by approximately 51.4%, while Cu-phendione reduced the *Leishmania*-macrophage interaction by 44.4% [44]. Oliveira and collaborators [25] found that THP-1 macrophages infected with *L. amazonensis* showed a drastic reduction in the AI after treatment with Ag-phendione and Cu-phendione, when compared to the untreated control. The IC_50_ values calculated for *L. amazonensis* amastigotes were 43 nM and 35 nM for Ag-phendione and Cu-phendione, respectively. In addition, Ag-phendione and Cu-phendione showed greater toxicity to *Leishmania* amastigotes compared to THP-1 macrophage cells, resulting in excellent SI selectivity index values: 42.0 for Ag-phendione and 43.4 for Cu-phendione.

Gp63 is released into the extracellular medium mainly by metacyclic promastigotes through exosomes [19]. The exosomes fuse with the macrophage membrane and release gp63 into the host cell cytoplasm, where it will act as a regulator of the cell’s functional activity, facilitating the internalization and survival of the parasite in the host cell [17,19,50]. Gp63 protects *Leishmania* from the hostile environment of the phagolysosome, since during the host-parasite interaction process it is capable of altering important macrophage signaling pathways, allowing the parasite to escape the host cell’s immune system [18,19,50]. Gp63 modulates the host cell’s immune response through different pathways. Within the macrophage, one of the main targets of gp63 is myristoylated alanine-rich kinase C (MARCKS) and MARCKS-related proteins (MRP), substrates for protein kinase C (PKC). PKC is involved in signal transduction associated with apoptosis, cell differentiation and proliferation, so that blockage of these pathways by gp63 directly affects the host cell defense system [17,22]. Gp63 is also responsible for activating protein tyrosine phosphatase (PTP) SHP-1, promoting JAK2 inactivation and modulating the production of nitric oxide and other inflammatory mediators [17,22,51]. In addition, gp63 acts on the macrophage translation system through cleavage of rapamycin (mTOR), a serine/threonine kinase that regulates a translational repressor [18,52]. Recently, a study evaluated through Western blotting the cleavage/degradation of host cell proteins that act as substrates for gp63. The authors infected bone marrow-derived macrophages (BMM) with wild type, *GP63*-defective Δ*gp63* mutants, or complemented Δ*gp63+GP63 L. major* metacyclic promastigotes and subsequently prepared cell lysates in the absence or presence of phen. The results showed that cell extracts from parasites mutated for gp63 or treated with the inhibitor were not able to cleave different targets previously described as substrates for gp63, such as: mTOR, VAMP3, NLRP3, PTP-PEST, SHP-1, Synaptotagmin XI and VAMP8. On the other hand, all these substrates were degraded when BMM was infected with wild-type parasites or Δ*gp63+GP63 L. major*, indicating that this metallopeptidase acts by inhibiting different host cell defense pathways [18].

Previous studies showed that phendione, Cu-phendione and Ag-phendione effectively inhibited the activity of secreted and membrane-associated gp63 in a dose-dependent manner, consequently promoting a reduction in the association index with peritoneal macrophages [44]. In this work, Ag-phendione presented an IC_50_ value equal to 2.16 µM for secreted gp63, while in *L. amazonensis* this value was 7.8 nM for promastigotes and 43 nM for amastigotes. Cu-phendione showed a very high IC_50_ value for secreted gp63 (IC_50_ = 163 µM), while for promastigotes and amastigotes these values were 7.5 and 35 nM, respectively, very close to the values observed for Ag-phendione. The differences between the IC_50_ values in the parasite and in the secreted gp63 as well as the similar effect of the compounds on the parasite points to the idea that Ag-phendione and Cu-phendione have a pleiotropic effect in the biology of the parasite. Corroborating this proposal, it was previously verified that the treatment of *L. amazonensis*, *L. infantum* and *L. braziliensis* promastigotes with these compounds promoted alterations in the mitochondrial metabolism as well as in the mitochondrial membrane potential [24,25]. Furthermore, *L. braziliensis* also showed an increase in the production of reactive oxygen species [24]. All these perturbations culminated in an increase in annexin-positive/propidium iodide-negative promastigotes as well as in DNA cleavage, indicating a death process similar to apoptosis [24,25]. A study that evaluated the effect of Ag-phendione and Cu-phendione on *P. aeruginosa* showed that the metal-based compounds interact with the DNA double strand through hydrogen bonds and hydrophobic and electrostatic interactions. Cu-phendione was also able to induce DNA relaxation and oxidative DNA damage, indicating that these compounds are capable of interacting with DNA, inhibiting the proliferation of the microorganism [34]. Altogether, these data indicate that Ag-phendione and Cu-phendione have more than one target in microbial cells. The action of Ag-phendione and Cu-phendione on gp63 may inhibit the parasite’s ability to modulate the macrophage defense system, favoring *Leishmania* survival, while the ability of the compounds to bind to DNA may block parasite proliferation.

## 4. Conclusions

The therapeutic arsenal for the treatment of leishmaniasis is limited and presents a series of limitations that hinder adherence and completion of treatment, such as different side effects, high cost and intravenous route of administration. Gp63 is an essential metallopeptidase in the establishment of the host infection and in the progression of the disease, being an attractive target for the development of new chemotherapeutics. In this sense, the search for new compounds that block the activity of this enzyme seems to be an interesting and promising path for the treatment of the disease. In the present study, we showed that phendione-derived compounds, Ag-phendione and Cu-phendione, can interact with gp63, blocking its proteolytic activity. Furthermore, promastigotes pre-treated with metal-based compounds showed less ability to interact with THP-1 macrophage cells, confirming the effectiveness of these compounds in blocking the establishment of infection. Collectively, our findings provide valuable insights into the potential use of these potent phendione-based complexes as part of an antivirulence therapy against *Leishmania*, providing new avenues for the development of effective treatments for leishmaniasis.

## Figures and Tables

**Table 1:** Energy values and intermolecular interactions provided by docking calculations.

Test Compounds	Total Interaction Energy (kcal/mol)	Hydrogen Bonds
**Phen**	−45.83	Ser_465_ Thr_467_ 2 H_2_O
**Phendione**	−39.75	4 H_2_O
**Ag-phendione**	−74.82	Ser_465_ Thr_467_ 6 H_2_O
**Cu-phendione**	−68.16	Ser_252_ Ser_465_ 5 H_2_O

**Table 2:** Kinetic parameters of the test compounds on the secreted gp63 activity.

Test Compounds	IC_50_ (µM)	K_i_ (µM)
**Phendione**	1494	744.8
**Ag-Phendione**	2.16	5.13
**Cu-Phendione**	163	27.05

## Data Availability

Not applicable.
